# A machine learning approach to wastewater treatment: Gaussian process regression and Monte Carlo analysis†

**DOI:** 10.1039/d4na01064g

**Published:** 2025-05-28

**Authors:** Nimra Nadeem, Zubair Khaliq, Abdulaziz Bentalib, Muhammad Bilal Qadir, Fayyaz Ahmad, Muhammad Wakil Shahzad, Abdulrahman Bin Jumah

**Affiliations:** a Department of Textile Engineering, National Textile University Faisalabad 37610 Pakistan; b Innovative Nanomaterials, Textiles and AI Solutions Research Group, National Textile University Faisalabad 37610 Pakistan; c Department of Materials, National Textile University Faisalabad 37610 Pakistan; d Department of Chemical Engineering, College of Engineering, King Saud University P. O. Box 800 Riyadh 11421 Saudi Arabia; e Department of Applied Sciences, National Textile University Faisalabad 37610 Pakistan fayyaztv94@gmail.com; f Department: Mechanical and Construction Engineering, Northumbria University Newcastle UK muhammad.w.shahzad@northumbria.ac.uk

## Abstract

This study aimed to analyze the application of Gaussian Process Regression (GPR) modeling to improve the accuracy of degradation response predictions in wastewater treatment. Three crucial factors, *i.e.*, catalyst (CFA–ZnF), oxidant (H_2_O_2_), and pollutant (MB) concentration, were selected to evaluate their impact on the response variable (degradation) using the GPR model. The range of factors was 5–15 mg/100 mL for CFA–ZnF, 5–15 mM for H_2_O_2_, and 5–15 ppm for MB concentration. The GPR model predicted the pairwise correlations of CFA–ZnF (0.4499, *p* = 0.0465) and H_2_O_2_ (0.4543, *p* = 0.0442) with degradation, which are moderately positive, while MB showed a weak negative correlation (−0.1686, *p* = 0.4774). Partial correlations also indicated strong positive correlations with degradation for CFA–ZnF (0.5143, *p* = 0.0290) and H_2_O_2_ (0.5180, *p* = 0.0277). The superiority of the GPR model was validated by comparing the Gaussian Process Regression Mean (RPAE value) of 0.92689 with the Polynomial Regression Mean (RPAE value of 2.2947). Besides, the simultaneous interpretation of the effects of the three predictors on the response variable was enabled using the GPR model, which is impossible when interpreting the polynomial regression model. Therefore, the GPR offers superior modeling, deeper insights, and reliable predictions, proving it to be a more sustainable and effective method for pollutant degradation in wastewater treatment than polynomial modeling.

## Abstract 2

This study integrates GPR with Monte Carlo simulations to enhance predictive accuracy in wastewater treatment, offering a more reliable, data-driven approach than traditional statistical or machine learning methods.

## Graphical abstract

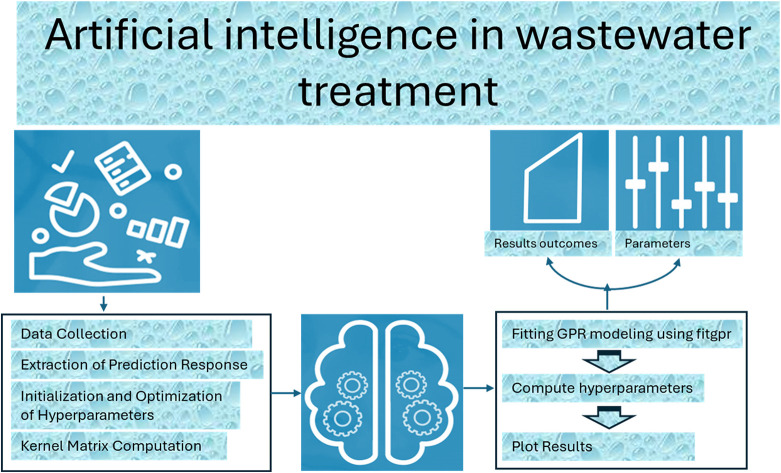

## 1 Introduction

The application of nanomaterials in photocatalysis has been studied extensively owing to their particle size and remarkable photo-driven response.^1–5^ The potential of various semiconductor materials as efficient photocatalysts has been exploited in wastewater treatment.^6–9^ The limitations associated with pristine semiconductor metal oxides can be overcome by compositing with value-added materials like coal fly ash (CFA).^10^ Various researchers have exploited the potential of CFA in wastewater treatment.^11^ Several researchers also exploited the sunlight-driven response of pristine semiconductor photocatalysts when compositing with CFA.^12,13^ CFA is a residual material left after coal combustion with a high content of alumina silicates and other metal oxides, serving as an excellent material to be used in compositing with other semiconductor metal oxides in various fields of energy and environments.^14^ Therefore, this is also recommended as a sustainable approach to waste management.

Artificial intelligence (AI) has recently enriched the field of environmental sciences, including wastewater treatment, owing to its ability to improve process efficiency, parameter optimization, and prediction of treatment outcomes. In the optimization and prediction of wastewater treatment, several machine learning tools are usually employed, including Artificial Neural Networks (ANN),^15^ Decision Trees (DTs),^16^ Random Forests (RFs),^17^ and Support Vector Machines (SVMs).^18^ Deep learning algorithms have improved the precision of data analysis, enabling real-time monitoring and adaptive control of treatment processes.^19^ For example, AI-driven hybrid models integrated with ML and computational fluid dynamics (CFD) have been promising models for optimizing electrochemical and photocatalytic treatment processes.^20^ These also provide sustainable approaches to wastewater management and reduce energy and chemical consumption during pollutant removal. AI-driven tools like extreme gradient boosting (XGBoost) are powerful algorithms with improved predictive accuracy by reducing bias and variance in complex data sets.^21^*K*-Nearest neighbors (KNN) help classify water quality data by analyzing the pollutant concentration patterns based on previous datasets.^22^ Recurrent neural networks (RNNs) are effective for time-series prediction in wastewater monitoring. This helps with real-time monitoring of treatment efficiency. Long short-term memory networks (LSTMs) are a type of RNN and ideal for the long-term prediction of wastewater treatment trends.^23^ Although these tools are capable of handling multidimensional data, the Gaussian Process Regression (GPR) model is superior.^24^ Numerous systematic reviews of Gaussian process regression (GPR) have demonstrated its capability to model interval prediction and handle missing and abnormal data effectively.^25^ Additionally, GPR has been found to possess the capacity to address challenges associated with high-dimensional and small-sample problems.^26–28^ GPR provides a probabilistic and non-parametric approach to capture non-linear and complex relationships with quantified uncertainty in prediction.^25,29^ The application of GPR modeling in photocatalytic degradation studies is of significant advantage over traditional polynomial regression modeling.^30^ Polynomial models are often constrained by their parametric nature and can be complex when interpreting non-linear relationships among various predictors. GPR modeling, on the other hand, provides a non-parametric, non-linear approach that can handle multidimensional datasets. The flexibility of GPR in capturing the complex interactions among predictors provides robust and accurate predictions with better reliability than the polynomial model.^31,32^

This research highlights the importance of GPR modeling in data predictions using several simulations and correlation methods with polynomial modeling. This research delivers a comprehensive understanding of the factors influencing degradation by analyzing the simultaneous interaction of the catalyst, oxidizing agent, and dye concentration. Predicting consumer-defined responses ensures that the outcomes are tailored to real-world applications, enhancing the practical relevance of the study. Furthermore, extensive simulations using Monte Carlo modeling provide robust statistical validation, highlighting the reliability and accuracy of the predictive models. Together, these elements underscore the significance of this work in advancing the field of wastewater treatment through sophisticated machine-learning approaches. Various machine learning tools have been employed in GPR modeling, including linear and partial correlations, partial dependence plots (PDPs), and 3D illustrations of comparative analysis of degradation predictions. This study involves running 100 unique simulations to assess the performance and reliability of the GPR model. In addition, the study also highlights the importance of Shapley values and feature importance metrics in interpreting the model predictions and analyzing the important contributing factors in degradation performance. The optimization segment leverages these insights to predict the optimal conditions of all factors, providing practical implications for all findings. Therefore, GPR offers superior modeling, deeper insights, and reliable predictions, proving it to be a more sustainable and effective method for pollutant degradation in wastewater treatment. The novelty of the proposed research lies in integrating GPR with Monte Carlo simulations to improve predictive accuracy in wastewater treatment modeling. This is a more reliable and data-driven optimization approach than conventional statistical and machine learning methods.

## 2 Methodology

### 2.1 Fabrication of influencing factor *X* (CFA–ZnF)

The facile hydrothermal approach was accessed for the fabrication of a CFA–ZnF composite (CFA–ZnFe_2_O_4_ (1 : 1)). Step 1 : 1 : 2 molar ratios (0.01 : 0.02) of Zn(NO_3_)_2_·6H_2_O and Fe(NO_3_)_3_·9H_2_O were mixed in 50 mL of distilled water. After mixing CFA (1.2 g) was added, followed by the dropwise addition of 50 mL of 8 M NaOH under constant stirring. The reaction was magnetically stirred for 1 h at 80 °C. The precursors were transferred into a Teflon-lined stainless steel autoclave reactor (250 mL) and placed in a heating oven for 24 h at 110 °C. After cooling, the obtained magnetic precursors were washed with distilled water and ethanol to remove impurities until neutralized. The magnetic CFA–ZnF was air dried at 70 °C.

### 2.2 Characterization

The CFA chemical composition was evaluated using X-ray fluorescence (XRF) spectroscopy. The chemical bonding and availability of surface functional groups in CFA–ZnF were analyzed using Fourier Transformed Infrared (FTIR) spectroscopy (PerkinElmer: spectrum 100: range 500–4000 cm^−1^). The crystal phase analysis of CFA–ZnF was performed using an X-ray diffractometer (XRD, Rigaku) coupled with a CuKα (*λ* = 0.154056 nm) radiation source at an operating voltage of 45 kV and a current of 40 mM. The XRD data were used to determine the crystallite size using the Debye–Scherrer equation. The morphology of the composite catalyst was determined using near-surface elemental analysis using a scanning electron microscope (SEM JSM-7000 F, ACCEL VOLT 15.0) equipped with energy-dispersive X-rays (EDX). To check the light response of the prepared catalysts, their energy band gaps were determined using a UV-UV-visible spectrophotometer (CECIL CE 7200). The magnetic characteristics of the composite introduced by the addition of ZnF were evaluated using a vibrating sample magnetometer (VSM) through an M–H curve under a 25 kOe applied magnetic field.

### 2.3 Conduction of the experiment (set-up details)

The photocatalytic dye degradation was performed under UV-254 nm in a digital chamber (ZamZam Micro Technologies ZM144W) with 8 UV lamps, each with a power of 18 W. The light intensity of the UV lamps was evaluated using a digital radiometer (UVX; UVP Analytic Jena) with a 254 nm probe. Methylene blue (MB: as a model pollutant: Factor *Y*) with a specific amount of CFA–ZnF was sonicated for a few seconds to attain good dispersion, followed by the addition of an oxidizing agent (H_2_O_2_: Factor *Z*). Then, the reaction mixture was placed in a UV chamber for 60 minutes. The details of the experiment are given in our previous publication.^11^ The design of the experiment was modulated using Design Expert-7 software, which used central composite design (CCD) under response surface methodology (RSM), with the range of the data set given in S-Table 1 (ESI).† The actual design generated by the software is given in Table 1, and the response is generated by experimentation.

**Table 1:** Design of experiment generated by RSM

Run	Factor 1	Factor 2	Factor 3	Response 1
	*X*: CFA–ZnF (mg L^−1^)	*Y*: H_2_O_2_ (mM)	*Z*: MB (ppm)	Degradation (%)
1	15	15	15	80.978
2	18.41	10	10	78.75
3	10	10	18.41	75.98
4	1.59	10	10	43.78
5	10	10	10	97.32
6	15	5	15	73.69
7	5	5	15	40.98
8	15	5	5	78.46
9	10	18.41	10	78.78
10	15	15	5	85.89
11	10	10	10	94.98
12	5	5	5	45.87
13	5	15	15	73.98
14	10	10	10	93.89
15	10	10	10	90.34
16	5	15	5	78.98
17	10	10	10	96.65
18	10	10	10	96.76
19	10	1.59	10	43.98
20	10	10	1.59	95.09

### 2.4 Machine learning

#### 2.4.1 Geometrical representation

**Fig. 1:**
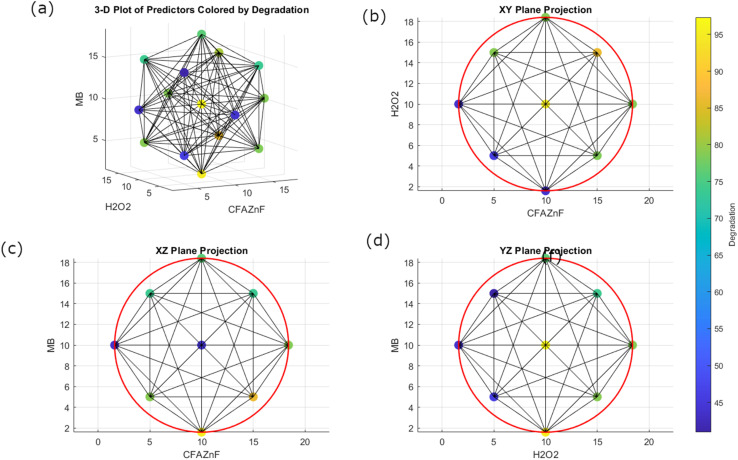
Geometrical representation of the model, 3D plot of all predictors colored by degradation (a), XY plane projection (b), XZ plane projection (c), YZ plane projection (d).

Geometric representation in data analysis and machine learning refers to visualizing data points in a geometrical space. Geometrical representation helps in understanding the relationships and patterns within the data. Fig. 1 shows the geometrical representation of the data set. The heatmap given at the right side of the figure helps in presenting the degradation response through visually mapping reaction parameter interaction using solar gradients, which helps in trend identification and optimization. Here, the relationship between the predictors (*X*: CFA-ZnF, *Y*: H_2_O_2_, and *Z*: MB) and response variable is given in the form of 3D (Fig. 1a)(interaction of all 3 predictors) and 2D (Fig. 1a–d) (interaction in terms of *XY*, *YZ*, and *ZX*) relationships. The 3D plot of predictors highlights where specific degradation values are prominent, indicating the regions in the predictor space associated with higher or lower degradation (presented by color variations). Meanwhile, in the 2D representation (*i.e.*, *XY*, *YZ*, and *ZX* plane projections), the plots represent the relationship of 2 predictors while keeping the third predictor at a certain constant value. It facilitates determining the dependency of 2 predictors in degradation response.

## 3 Results and discussion

### 3.1 Characterization

XRF analysis confirms the F-type of CFA with the Al_2_O_3_ + SiO_2_ + Fe_2_O_3_ cumulative composition greater than 70%. The successful insertion of ZnF into CFA has been confirmed by XRD analysis as the composite catalyst showed characteristic peaks of ZnF (JCPDS 002-4496) including the characteristic peaks of CFA (*i.e.*, quartz (Q) and mullite (M)). The Debye–Scherrer equation was used for the calculation of crystallite size, which was found to be 29.43 nm for CFA–ZnF. The identification of surface functional groups in CFA–ZnF by FTIR showed a stretching vibrational mode of Fe–O around ∼621 cm^−1^. The peaks at ∼820 cm–820 cm^−1^ and ∼970 cm^−1^ are attributed to the stretching and bending vibrational modes of Al–O and Si–O bonds, respectively. The comprehensive details of all the peaks present in the FTIR spectra of CFA–ZnF are discussed elsewhere. Considering the result of surface morphological analysis *via* SEM, the irregularity in the composite structure has been observed due to the insertion of CFA into pristine ZnF NPs. This amorphous structure modification is responsible for better adsorption followed by effective photocatalytic degradation of pollutant molecules. The high roughness in the catalyst surface provides better fractional dimensions, resulting in improved interaction between the adsorbent and adsorbate. The EDX results confirmed the presence of essential elemental components in CFA–ZnF, including C, O, Al, Si, Zn, Fe, and Ca. The characterization results can be found in the ESI document (Fig. S-5)† as well as in previously published work^11^

The magnetic response of CFA–ZnF is confirmed by VSM analysis with a saturation magnetization (*M*_s_) value of 4.18005 ± 0.04901 emu g^−1^ for CFA–ZnF. A considerable reduction in the *M*_s_ value has been observed after the addition of CFA into ZnF (*i.e.*, from 34.01834 ± 0.24815 emu g^−1^ for ZnF to 4.18005 ± 0.04901 emu g^−1^ for CFA–ZnF). This is ascribed to the fact that the CFA contains only ∼15 wt% magnetic constituents. When an equal amount of CFA is added into ZnF. The elemental composition of CFA–ZnF was evaluated using XPS analysis. The results clearly showed a high-intensity C 1s peak attributed to the existence of CFA. Other characteristic peaks corresponding to ZnF were also successfully detected.

The optical response of CFA–ZnF was studied using UV-Vis spectroscopic analysis. The UV-Vis scan data of CFA–ZnF were used to compute the energy bandgap (*E*_g_) using the Tauc plot method. The *E*_g_ of CFA–ZnF was 3.10 eV, suggesting the better response of the composite catalyst under UV irradiations. Therefore, UV-254 nm lamps were used for the estimation of photocatalytic response by CFA–ZnF. The results of all the above characterization analyses with further details are presented in our previous publication.

### 3.2 Linear and partial correlations

**Fig. 2:**
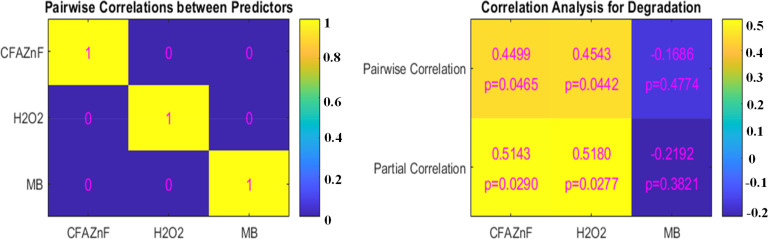
Correlation analysis: pairwise correlations within predictors (left panel). Pairwise and partial correlations of predictors with degradation (right panel).

The linear and partial correlations help understand how the predictors alone and collectively influence the degradation process (*i.e.*, response variable). The given *p*-value represents the statistical importance of correlations where a *p*-value less than 0.05 indicates a significant relationship. Fig. 2 illustrates the heatmap of pairwise correlation between all three predictors, *i.e.*, (CFA–ZnF *X*, H_2_O_2_*Y*, and MB *Z*). The heatmap shows that correlation values range from −1 to 1. A perfect positive correlation has been observed with the predictors (yellow area with a value of 1). This showed that all predictors *X*, *Y*, and *Z* showed a significant influence (individually) on the response variable. No correlations between CFA/ZnF and H_2_O_2_, CFA/ZnF and MB, and H_2_O_2_ and MB have been observed. Similarly, the pairwise and partial correlations of the predictors are presented in Fig. 2 (right side). The pairwise correlation of CFA–ZnF and H_2_O_2_ with degradation was found to be 0.4499 (*p* = 0.0465) and 0.4543 (*p* = 0.0442), reflecting a moderate positive correlation. Meanwhile, the pairwise correlation of MB with degradation is −0.1686 (*p* = 0.4774), indicating a weak negative correlation. A similar trend of partial correlation with degradation was observed; for example, CFA–ZnF and H_2_O_2_ present a strong positive correlation with degradation (when controlling another predictor) with the values of 0.5143 (*p* = 0.0290) and 0.5180 (*p* = 0.0277), respectively. A weak negative partial correlation of MB (−0.2192, *p* = 0.3821) with degradation was observed.

Hence, CFA–ZnF and H_2_O_2_ showed a moderate pairwise correlation with degradation when considered individually while MB exerted a slightly negative effect. The positive partial correlation of CFA–ZnF and H_2_O_2_ with degradation, while considering the impact of other predictors, indicates that these two variables independently contribute towards the degradation process. Meanwhile, MB exerts no significant effect on degradation, which is advantageous in terms of pollutant concentration. This suggests that the dose or concentration of MB is not a significant factor that contributes towards degradation processes up to a certain limit.

### 3.3 Machine learning model and response surface methodology

**Fig. 3:**
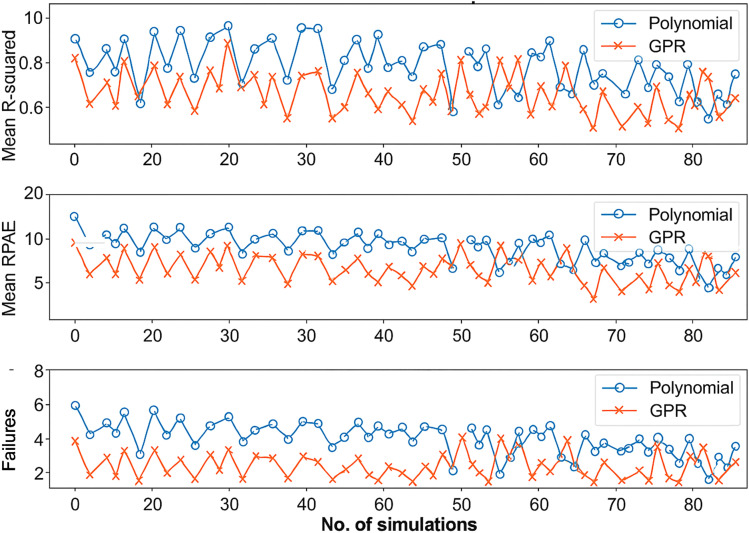
Model performance comparison: *R*-squared values, RPAE and no. of failures for polynomial and GPR models over 100 simulations.

Fig. 3 presents a comparative analysis of two model performances, *i.e.*, between the polynomial model and Gaussian Process Regression (GPR) over 100 simulations (Monte Carlo simulation). Monte Carlo simulation serves as a robust statistical tool that enhances the reliability of our predictive model by repeatedly sampling the input space (100 simulations in our case). Monte Carlo methods help to generate a probabilistic distribution of performance metrics (*e.g.*, *R*-squared, RPAE, and failure rates), thereby offering insights into the variability and risk associated with predictions. The approach allows us to assess the stability and robustness of the Gaussian Process Regression (GPR) model under different experimental conditions, which is crucial for optimizing the treatment process. Repeated simulations help validate that the GPR model's superior performance over traditional polynomial regression is consistent, reinforcing the practical reliability of the model in complex, multidimensional datasets such as those encountered in wastewater treatment. Wan and colleagues^33^ developed a water quality prediction model for papermaking wastewater treatment by combining Gaussian Process Regression with deep learning methods.^33^ Monte Carlo simulation was employed to quantify model uncertainties and enhance prediction robustness. Li and coworkers^29^ utilized Bayesian neural networks in material property prediction, incorporating Monte Carlo methods for uncertainty quantification. Their approach underscores how Monte Carlo sampling can be effectively coupled with machine learning to manage the intrinsic uncertainties in complex datasets.

The performance metrics include *R*-squared, RPAE (Relative Prediction Absolute Error), and failures. The coefficient of determination (*i.e.*, *R*-square) determines the percentage of variance in the dependent variable, which is predictable from the independent variable. The blue circle presents the performance of the polynomial model, whereas the red crosses represent the performance of the GPR model. As reflected, the *R*^2^ values in the GPR model are consistently higher than those of the polynomial model across most simulations (Fig. 3a). The GPR model demonstrates stable and higher *R*^2^ values presenting the ability of the model to explain a larger proportion of variance in data as compared to the polynomial model. The relative prediction absolute error (RPAE) measures the prediction error relative to the actual value. In the polynomial model, the RPAE values vary significantly with relatively higher errors compared to the GPR model, which has relatively accurate predictions compared to the polynomial model over many simulations (Fig. 3b). The GPR model consistently achieves lower RPAE values, highlighting its superior prediction accuracy. Failures in the model present how many times the model prediction errors exceed its threshold limit. As depicted, the GPR model consistently showed fewer failures than the polynomial model across simulations (Fig. 3c). It represents the higher reliability of the GPR model with less chance of significant prediction errors.

The consistent performance improvements of the GPR model over the polynomial model across different metrics validate the effectiveness and robustness of the GPR approach. This makes GPR more suitable for modelling and predicting complex data patterns, especially where accuracy and reliability are critical.

**Fig. 4:**
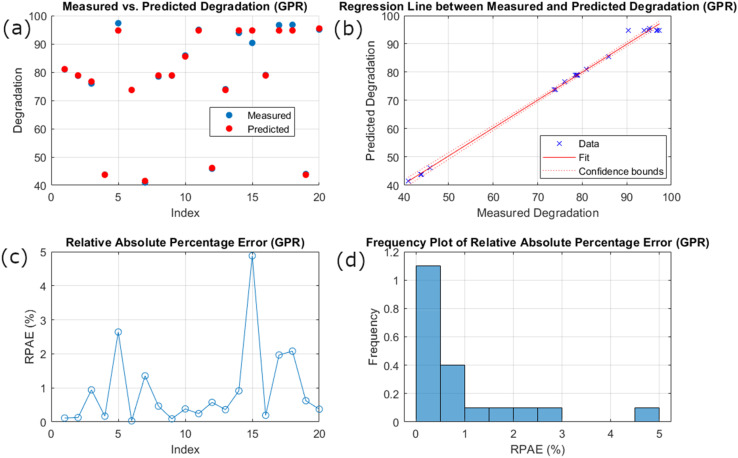
GPR model performance: (a) scatter plot of measured *vs.* predicted degradation. (b) Regression line between measured and predicted degradation. (c) Line plot of RPAE. (d) Frequency histogram of RPAE.

Fig. 4 displays the prediction of degradation analysis by the GPR model in four distinct plots. The scatter plot represents the measured degradation values (red circles) and predicted degradation values (blue circles) for several indices. The better agreement of both values suggests the proficiency of the GPR model in predicting the underlying data pattern, demonstrating extremely high accuracy in prediction (Fig. 4a). Fig. 4b represents the regression line between predicted and measured values. The figure proposes the model's reliability and high precision. The red regression line follows the close diagonal with a strong linear relationship. Similarly, narrow confidence bounds further highlight the model's consistency. The Relative Absolute Percentage Error (RPAE) for the individual index is presented in the Fig. 4c plot. On average, the RAPE values remain low with narrow fluctuation, highlighting the model's prediction accuracy. The RAPE histogram with frequency distribution (Fig. 4d) showed most of the values near 0–1%. The high data concentration point indicates that most of the predictions are accurate with only a few instances showing high errors.

On average, these plots present the GPR model's superior performance in predicting degradation. The alignment between measured and predicted values, the strong linear relationship, and the low and concentrated RPAE values all demonstrate the model's high accuracy, precision, and reliability. Therefore, the GPR model proves to be a highly effective and robust tool for predicting degradation, standing out as a superior choice to other models.

**Fig. 5:**
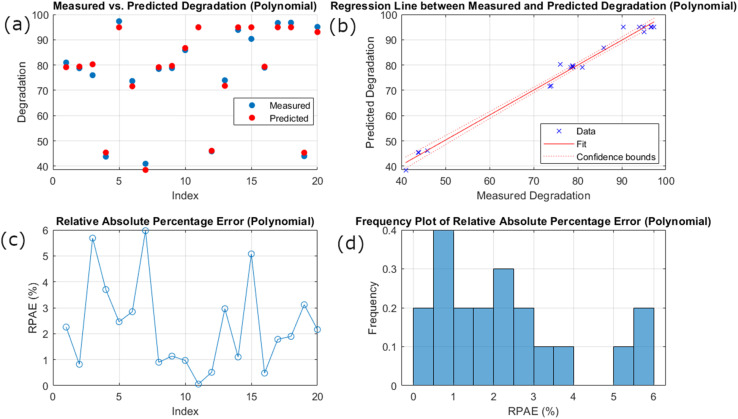
Polynomial model performance: (a) scatter plot of measured *vs.* predicted degradation. (b) Regression line between measured and predicted degradation. (c) Line plot of RPAE. (d) Frequency histogram of RPAE.

Fig. 5 illustrates the assessment of polynomial model performance in predicting the response variable (degradation). In the figure, the measured values are denoted with red dots, and predicted degradation values are represented by blue dots. The proximity between the measured and predicted values is evident from the graph. However, some discrepancies suggest room for improvement in model prediction. While comparing the results of the GPR model of predicted and measured values, in the GPR model, the variation is even smaller, ensuring the better performance of the model in predicting the degradation results. The plot of the regression line between measured and predicted degradation suggests that, as compared to the GPR model, the polynomial model shows less ‘fit’ and ‘confidence bounds’ with a comparatively high prediction uncertainty range. By examining the RAPE plot, the polynomial model exhibits varying percentage errors around several indices with different points of relatively high errors. In the GPR model, consistently low percentage errors with minor spikes predict better uniformity and small prediction errors. This trend is strengthened by the frequency plot of relatively absolute percentage error, where the broad spread of RAPE values is present in a polynomial model with considerable high-frequency error. Meanwhile, the frequency distribution of the GPR model is intense at the lower end of the RAPE scale, suggesting the relatively low prediction error in most of its matrices and smaller instances of high errors.

The comparison of the polynomial and the GPR model suggests that the GPR model constantly outperforms the polynomial model in terms of model reliability, error consistency, and prediction error. The relative proximity of measured *vs.* predicted values, tighter clustering around the regression line, and fewer and uniform errors in the GPR model suggest its superior performance over the polynomial model. Similarly, narrow confidence bounds confirm the reliability of the GPR model. These findings endorse the high accuracy and suitability of the GPR model in predicting degradation, making it a highly recommended approach for precise and reliable predictions.

The Gaussian Process Regression (GPR) approach models an unknown function as a Gaussian process defined by a mean and a covariance (kernel) function, implying that any finite collection of function values follows a joint Gaussian distribution.^34^ This assumption underpins the model's ability to quantify uncertainty in predictions, making it especially attractive for applications such as wastewater treatment, where decision-making benefits from probabilistic estimates. However, GPR assumes that the noise in the data is independently and identically distributed and follows a Gaussian distribution, which may not hold true when the data contain extreme values or outliers; such deviations can lead to biased predictions and misestimated uncertainties. Moreover, while GPR excels in capturing non-linear relationships, its performance can deteriorate in high-dimensional settings—a challenge often referred to as the “curse of dimensionality”—where the kernel may struggle to encapsulate the complex structure of the data without additional dimensionality reduction or regularization techniques.^25^ These limitations highlight the need for careful data preprocessing and model validation, ensuring that the assumptions inherent in GPR are reasonably met, especially in environmental applications where data variability is common.^26,31^

**Fig. 6:**
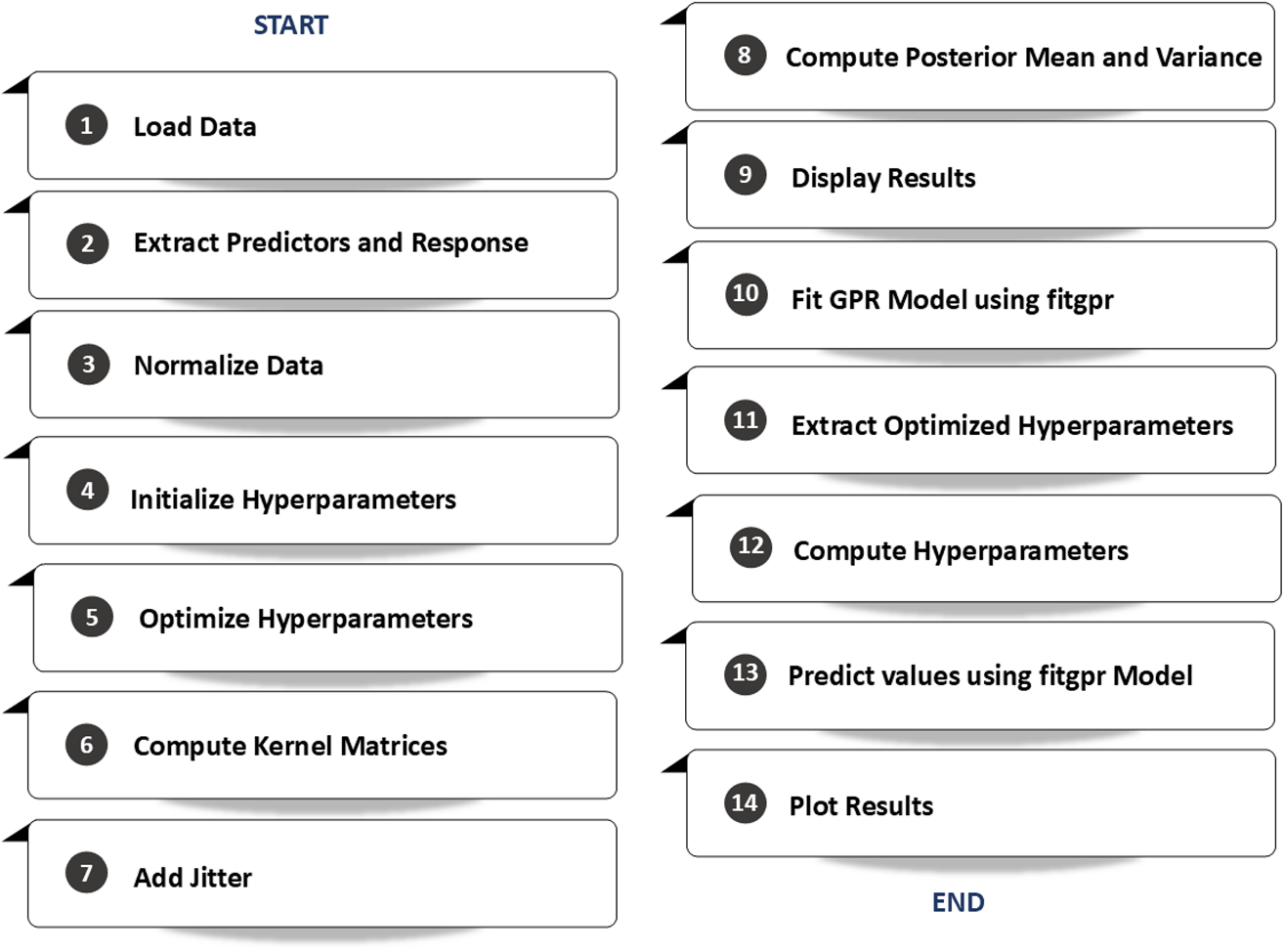
A stepwise schematic for Gaussian process regression with ARD kernel and hyperparameter optimization.

Gaussian Process Regression (GPR) is a non-parametric Bayesian approach that models complex, nonlinear relationships by placing a probability distribution over possible functions that can explain the observed data, thus providing point estimates and associated uncertainty measures. This probabilistic framework is particularly valuable in applications like wastewater treatment, where understanding the uncertainty in predictions can guide risk assessment and process optimization.^33^ Using kernel functions, GPR captures the similarity between data points and flexibly models intricate interactions among multiple predictors without assuming a fixed functional form. This inherent flexibility makes GPR especially effective when working with limited or noisy datasets, as it can adapt to the complexity of the data while mitigating overfitting issues.^29^ Furthermore, the built-in hyperparameter optimization process fine-tunes parameters that govern the kernel's behavior, enhancing both the fit's accuracy and the reliability of uncertainty estimates.^35^ This is crucial when predictions must be validated through Monte Carlo simulations, demonstrating that GPR consistently outperforms traditional methods like polynomial regression by providing more robust predictions and detailed confidence intervals.^29,33^ Additional studies have reinforced GPR's efficacy in complex environmental systems; for example, research by Hvala and Kocijan^32^ highlighted its capability in a hybrid mechanistic/GPR model for predicting wastewater treatment plant effluent, underscoring the method's practical relevance in real-world applications. These attributes—the ability to model nonlinearity, quantify uncertainty, and optimize hyperparameters—underscore why GPR is an indispensable tool in advanced predictive modeling, particularly in fields that demand high reliability and detailed risk assessment (Fig. 6).
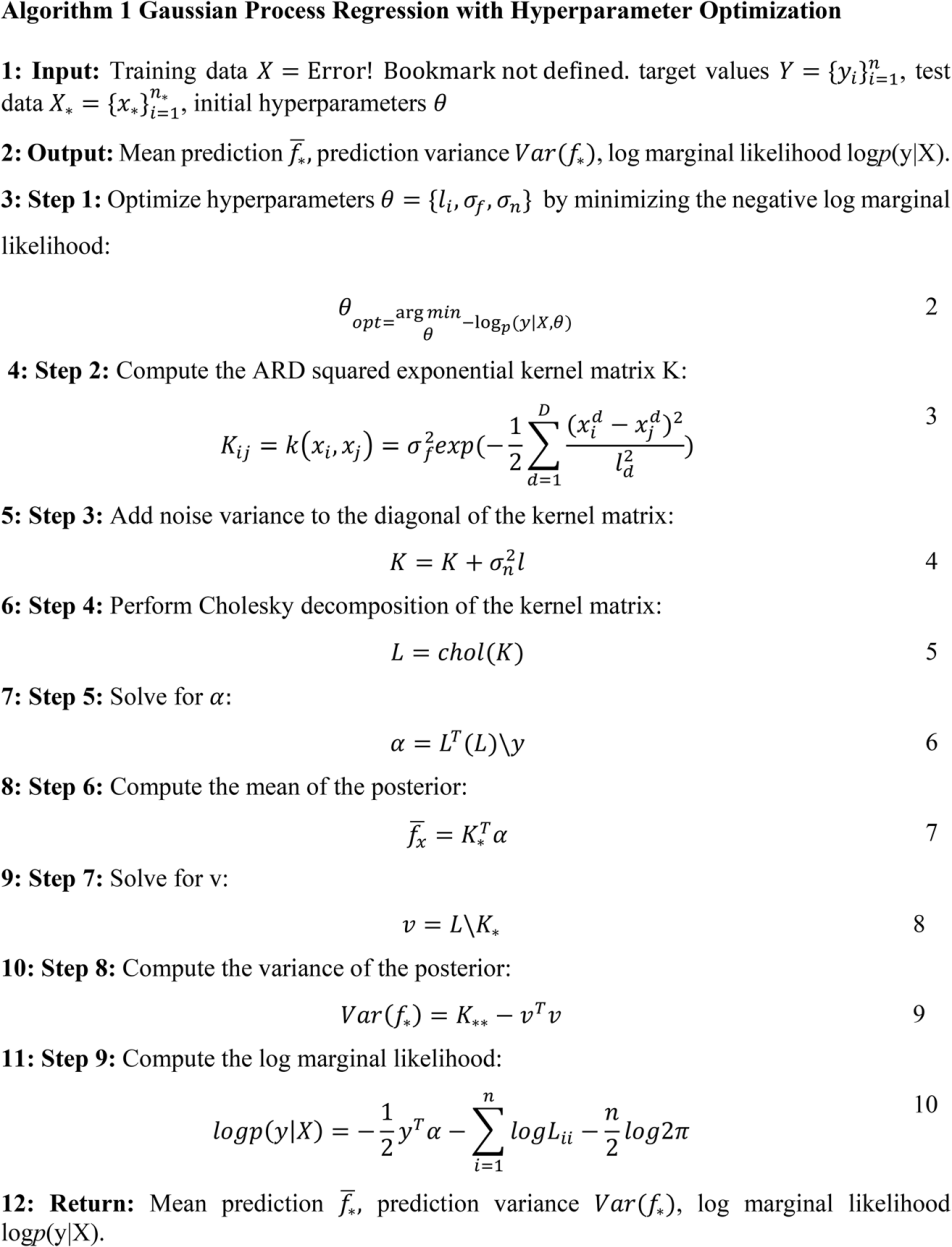


#### 3.3.1 Linear regression model

Degradation ∼1 + *β*_1_CFAZnF + *β*_2_H_2_O_2_ + *β*_3_MB + *β*_4_CFAZnF^2^ + *β*_5_H_2_O_2_^2^ + *β*_6_MB^2^ + *β*_7_CFAZnF × H_2_O_2_ +*ε*

Here

• *β*_1_, *β*_2_, *β*_3_, *β*_4_, *β*_5_, *β*_6_, and *β*_7_ are coefficients of variables

• *ε* is the error term

#### 3.3. 2Estimated coefficients

**Table:** 

Term	Estimate	SE	tStat	*p*-Value
(Intercept)	−67.309	6.2527	−10.765	1.6061 × 10^−7^
CFAZnF	13.789	0.68111	20.244	1.2103 × 10^−10^
H_2_O_2_	13.776	0.68111	20.225	1.2237 × 10^−10^
MB	1.5778	0.57105	2.763	0.017183
CFAZnF_sq	−0.4599	0.027655	−16.63	1.1882 × 10^−9^
H_2_O_2__sq	−0.45827	0.027655	−16.571	1.2377 × 10^−9^
MB_sq	−0.11675	0.027655	−4.2218	0.001185
CFAZnF_H_2_O_2_	−0.25696	0.037124	−6.9217	1.6013 × 10^−5^

#### 3.3.3 Model summary

• Number of observations: 20

• Error degrees of freedom: 12

• Root mean squared error: 2.63

• *R*-Squared: 0.988

• Adjusted *R*-squared: 0.981

• *F*-Statistic *vs.* constant model: 141

• *p*-Value: 1.45 × 10^−10^

The table shows the results of regression analysis. The estimated coefficient represents the change in the independent variable for a one-unit change in the predictor, keeping the rest of the predictors constant. For example, the coefficient for CFAZnF is 13.789, which means that a one-unit increase in the value of CFAZnF results in a 13.789-unit increase in the response variable, *i.e.*, degradation efficiency, by keeping all other factors constant. The standard error (SE) suggests precision in the estimation of the estimate coefficient, as SE is relatively quite small. The value of tStat for CFAZnF is quite large (*i.e.*, 20.244), presenting potential impact on dependent variables. Similarly, the significance of the model term CFAZnF is also evident from the *p*-value, *i.e.*, extremely small (1.2103 × 10^−10^).

### 3.4 Partial dependence plots (PDPs)

**Fig. 7:**
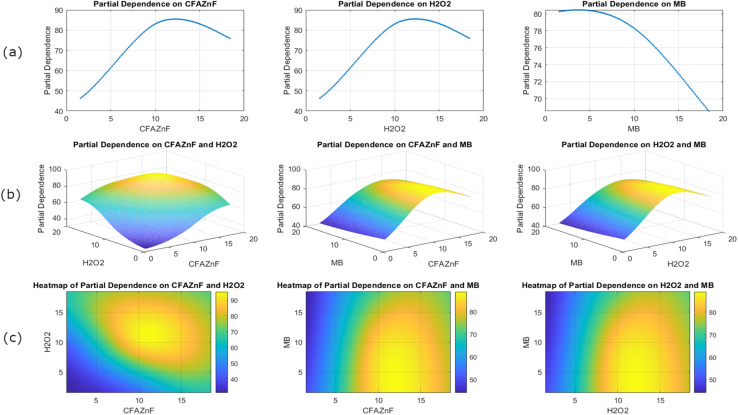
Visualizations depicting the partial dependence of CFAZF, H_2_O_2_, and MB on model predictions, showing individual (a) and interaction effects through 2D and 3D plot (b), 3D plot top view (c).

Fig. 7 presents the PDPs and heatmaps for CFA–ZnF, H_2_O_2_, and MB. Here, the marginal effect of individual features on percentage degradation is analyzed. The plots in Fig. 7a present a partial dependence on a single feature. These graphs show the marginal effect of individual features on the model's prediction. For features CFA–ZnF and H_2_O_2_, a non-linear relationship has been observed after the optimum achievement of response. Both these features positively affect the response value up to a specific limit, after which a decrease in response is observed. This defines the optimum concentration of CFA–ZnF and H_2_O_2_, where the maximum influence of both factors effectively achieves the best result for degradation values. Above the optimum point, the effectiveness weakens due to the saturation effect, leading to a decrease in the response variable. The decrease in response variable above the certainly optimized point is also attributed to the overlapping of catalyst active surfaces, causing the agglomeration of nanoparticles and resulting in low dye degradation. Considering the impact of MB on degradation, the negative impact is above a specific value. At low MB levels, the degradation is maximum and starts to decrease above the optimized concentration. This suggests that the high MB dosage (above a certain limit) is unsuitable for achieving a high response value. Therefore, managing the concentration of MB is crucial to avoid its adverse impact on treatment efficiency.

The 3D surface plots in Fig. 7b illustrate the pairwise interaction between features. For example, the interaction of CFA–ZnF and H_2_O_2_ is complex with a nuanced prediction pattern as compared to the facile prediction when considered individually. Similarly, other interactions of CFA–ZnF and MB, MB, and H_2_O_2_ highlight the area of antagonism and synergy, which is important in understanding the mutual influence on the response variable.

The heat maps presented in Fig. 7c provide a more intuitive presentation of mutual effects. Here, the combined effect of two features on degradation is presented using a color gradient, with warmer colors representing high degradation values. From this presentation, the most useful and most effective feature combination can be selected, aiding in model optimization and feature engineering strategy.

### 3.5 Comparative analysis of degradation prediction in 3D models

**Fig. 8:**
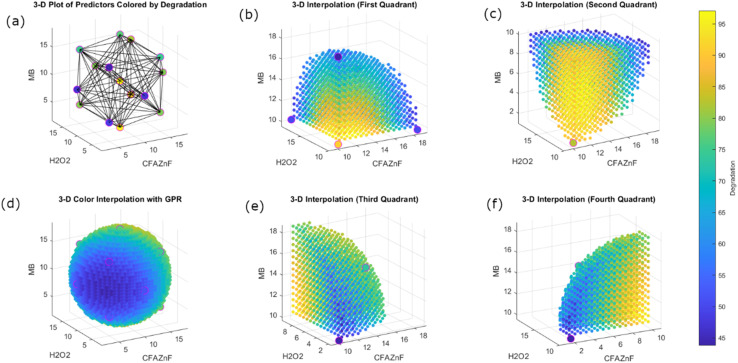
Degradation 3-D presentation with respect to predictors: (a) 3-D geometric representation of data. 3-D heat maps of degradation in four quadrants are shown (b, c, e and f). (d) 3-D full view of the heat map of degradation.

Fig. 8 illustrates the efficiency of the GPR model for a system consisting of three variables and their cumulative impact on degradation values. This model is not achievable by a polynomial model where the three-factor response can be studied simultaneously. The 3D plot of predictors colored by degradation in Fig. 8a presents the color-coded degradation for clarity. The successive plots present a series of 3D interpretations, each consisting of different quadrants that present the predicted degradation distribution across spatial parameters. The color gradient in 3D plots represents varying levels of dye degradation from blue to yellow. On average, the core of the individual quadrant shows the highest degradation value. The consistent gradient and smooth transition in the first (Fig. 8b), second (Fig. 8c), third (Fig. 8e), and fourth quadrants (Fig. 8f) suggest the strong capability of the GPR model in capturing the underlined data accurately. To further illustrate the predicted degradation along the entire parameter space in a comprehensive way, 3D color interpolation with GPR is provided, which shows the model's robustness in predicting response. The spherical representation provides a well-distributed interpretation, providing the GPR model's ability to generalize data well across the different regions. This is in stark contrast to polynomial models, which often struggle with overfitting and underfitting in complex, multi-dimensional spaces. The GPR's ability to present complex relationships more effectively than the polynomial model makes this model a more suitable choice for this type of application study, with more precise and reliable predictions crucial for control and optimization. The GPR model demonstrates superior interpolation and prediction accuracy compared to polynomial models, highlighting its robustness in modeling complex degradation patterns.

### 3.6 Shapley values

**Fig. 9:**
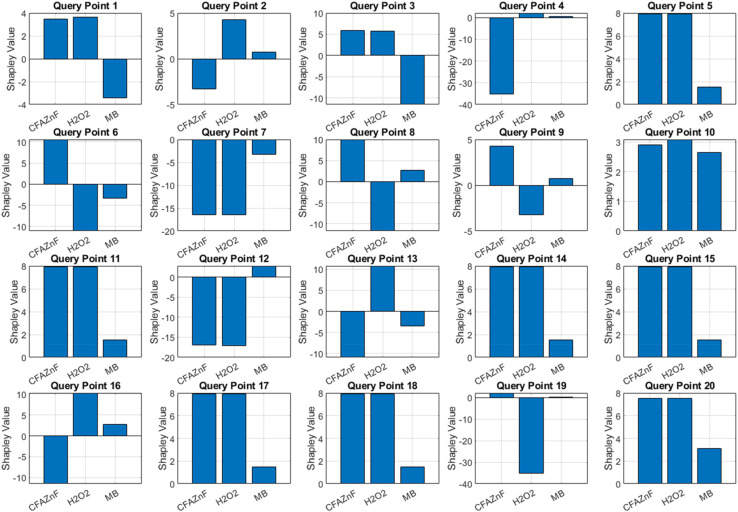
Bar charts illustrating SHAP values for CFAZF, H_2_O_2_, and MB across 20 query points, highlighting the varying contributions of each feature to the model's predictions.

The Shapley values provide visualization of 20 different query points, which helps in understanding the role of every feature in the model's prediction in particular instances. The Shapley bar chart presents the influence of each feature on the prediction for each query point (Fig. 9). Positive Shapley values suggest a high prediction of features and negative values suggest a lower impact. For example, in query point 1, CFA–ZnF and H_2_O_2_ positively contribute towards the prediction, while MB has a negative effect. Similarly, in query point 2, H_2_O_2_ has a negative impact, reducing prediction. The diverse Shapley values across 20 query points showed the relationship of each feature at a particular instance. This diversity of the model indicates the reliability and context dependency of each feature. For example, in query points 6 and 8, CFA–ZnF exhibits a consistently high positive Shapley value, presenting its significant contribution in these cases. The chart also helps in predicting the interactions among all features. For example, in query point 11, CFA–ZnF and H_2_O_2_ possess high Shapley values, predicting their positive synergistic effect at a certain level. Contrarily, in query point 3, MB exhibits a high negative Shapley value at specific positive values of CFA–ZnF and H_2_O_2_. Similarly, other query points can be addressed. On average, in most of the query points, CFA–ZnF showed a positive contribution, indicating that it plays a key role in the degradation process. Moreover, CFA–ZnF offers a diverse condition range, suggesting its effectiveness in degradation. Meanwhile, H_2_O_2_ offers both the positive and negative Shapley values around different query points, indicating its dual role in the degradation process. This also suggests that its effectiveness may also depend on the interaction and concentration with another component. The high negative values in specific query points are attributed to the inhibitory effect of H_2_O_2_ under certain conditions, which has been verified practically. Considering the MB Shapley values, its negative role across many query points suggests that it may negatively impact the degradation study under certain conditions. The negative role of MB in most of the prediction results is attributed to the fact that the degradation process may not be very effective when MB concentration varies too much. However, under optimized experimental conditions (including that of MB), 98% degradation can be achieved.

### 3.7 Feature importance

**Fig. 10:**
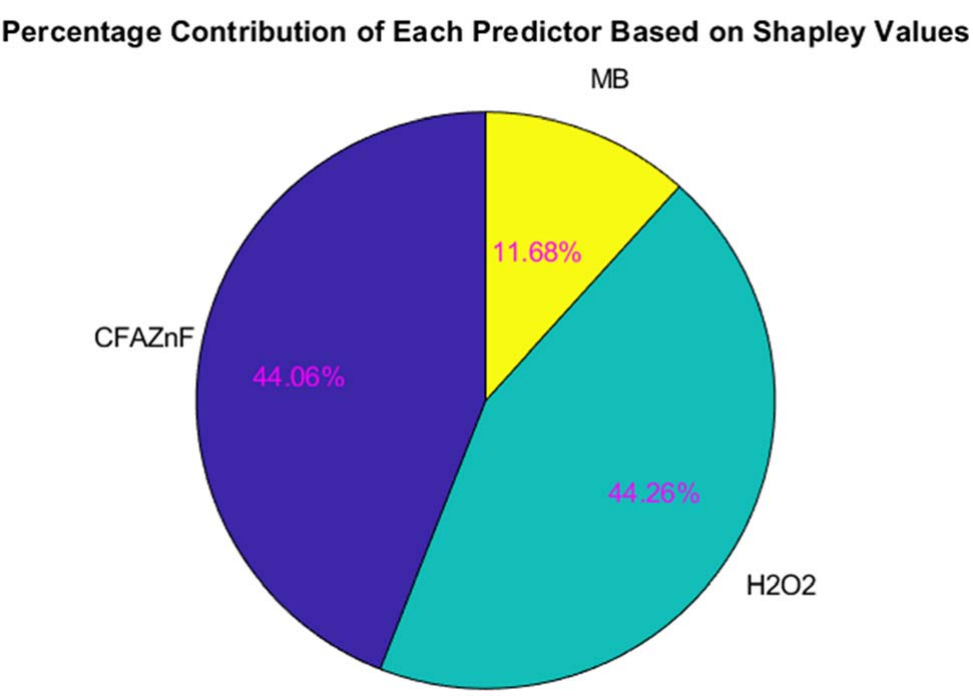
Pie chart showing the percentage contribution of CFAZF (44.06%), HOQ2 (44.26%), and MB (11.68%) to the model's predictions for degradation.

The importance of Shapley values is further supported by the pie chart representation (Fig. 10). The pie chart presents a relatively clear picture of the importance of individual features based on Shapley values. The graph presents the highest and almost equal contribution of CFA–ZnF and H_2_O_2_ (CFAZnF: 44.06% and H_2_O_2_: 44.26%) in the model's prediction. Therefore, both these features can be considered as the driving features in the model's output. The substantial influence of these two features suggests that the model highly relies on CFA–ZnF and H_2_O_2_ to realize accurate predictions. Meanwhile, MB offers a significantly smaller contribution as compared to the other two features. This suggests that although MB affects the model's prediction, its role is less critical as compared to CFA–ZnF and H_2_O_2_. The percentage variations also suggest that almost equal contributions of both CFA–ZnF and H_2_O_2_ are required in predicting the degradation response, while MB has a more specialized role (*i.e.*, high dependency on concentration). Considering the relative contribution of individual features can help in scheming a better, efficient, and cost-effective treatment process.

### 3.8 Optimization

**Fig. 11:**
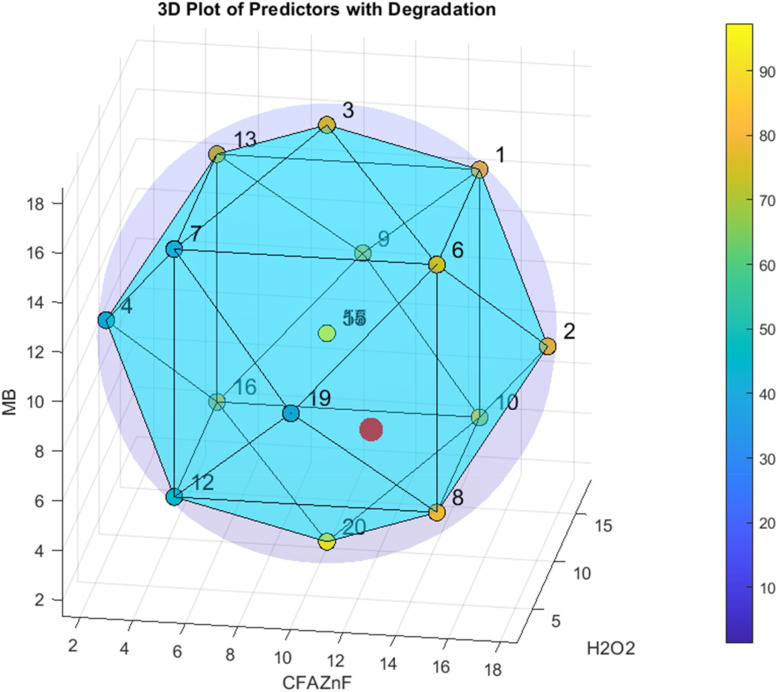
3D heat coloring map of degradation with respect to CFA–ZnF, H_2_O_2_, and MB. Maximum degradation is at the red dot.

Fig. 11 represents the #D illustration of the relationship between predictors (*i.e.*, CFA–ZnF, H_2_O_2_, and MB) and response (*i.e.*, degradation). The blue-to-yellow color scale presents the degradation values from lower to higher, respectively. The data point represents the combined effect of these predictors on degradation with the specification of degradation values denoted by a specific color. The numbering of data points is the specific experimental condition with the color gradient representing the degradation level. The red dot represents the point of maximum degradation at the optimized level of predictors and the point close to red (*i.e.*, yellow) represents high levels of degradation. The central point 5 with a green color represents a medium degradation value proposing the mid-range for all three predictors on average degradation. The data point with light blue surfaces illustrates the interactions among predictors and it helps in understanding the impact of change in the concentration of one predictor on another predictor while keeping the other predictor constant. The contour lines help analyze the gradient and transition of degradation values across various combinations. This plot aids in categorizing the optimum conditions of all predictors in achieving maximum degradation. By carefully analyzing the color shift region towards yellow, the researchers can find out the best combination of all predictors. It also helps in validating the predictive models. The model is suitable for designing the customer-defined response values as the model accuracy is confirmed by the perfect alignment of experimental data with that of predicted surfaces.

## 4 Conclusion

This research underscores the pivotal role of advanced machine learning techniques in optimizing wastewater treatment degradation processes using a CFA–ZnF composite catalyst. By analyzing the simultaneous interaction of three key predictors—catalyst type, oxidizing agent, and dye concentration—this study provides a nuanced understanding of their combined effects on degradation efficiency. The Gaussian Process Regression (GPR) model demonstrates superior performance in capturing intricate, non-linear relationships. The consumer-defined response prediction ensures that the developed models are highly applicable to real-world scenarios, enhancing practical utility. Additionally, 100 Monte Carlo simulations bolster the statistical robustness of the findings, offering a comprehensive validation framework and elucidating the variability and uncertainty associated with the predictors. This approach allowed for exploring various reaction conditions, providing a more comprehensive understanding of the degradation process. Tailored reaction conditions were identified, maximizing degradation efficiency by leveraging the predictive power of the GPR model. This consumer-defined approach ensures theoretical robustness and practical relevance, offering valuable insights for real-world applications in wastewater treatment. Overall, this study advances the field by demonstrating the efficacy of sophisticated machine learning methodologies in optimizing and predicting degradation outcomes, holding substantial promise for developing more efficient, reliable, and consumer-responsive wastewater treatment processes. The integration of GPR modeling, consumer-defined response prediction, and Monte Carlo simulations offers a powerful framework for addressing complex environmental challenges, contributing to sustainable wastewater management. These findings pave the way for future research and practical applications in environmental management and sustainability, highlighting the potential of advanced machine learning techniques in environmental science. The combined use of GPR and Monte Carlo analysis advances data-driven approaches in environmental remediations, particularly in wastewater treatment (in this research), therefore providing more efficient and cost-effective remediation technologies.

## Conflicts of interest

There are no conflicts to declare.

## Supplementary Material

NA-OLF-D4NA01064G-s001

## Data Availability

The data supporting this article have been included as part of the ESI.†
