# Supplementary material for: A machine learning approach to wastewater treatment: Gaussian process regression and Monte Carlo analysis
Source: Nanoscale Adv. 2025 May 28;7(14):4436–49. doi: 10.1039/d4na01064g (PMC12150233; doi:10.1039/d4na01064g)
Supplement: NA-007-D4NA01064G-s001 [file NA-007-D4NA01064G-s001.pdf]

Supplementary Information (SI) for Nanoscale Advances.
This journal is © The Royal Society of Chemistry 2025

Supplementary Information

A Machine Learning Approach to Wastewater Treatment: Gaussian Process Regression and Monte Carlo Analysis

Table S-1: Range of parameters given to CCD under RSM

	**Name**	**Unit**	**Low**	**High**
A	CFA-ZnF	Mg/100 mL	5	15
B	H_2O_2	mM	5	15
C	MB	ppm	5	15

Table S-2 ANOVA results of the quadratic model for degradation of MB using CFA-$ZnFe_2O_4$

**Source**	**Sum of Squares**	**df**	**Mean Square**	**F-value**	**p-value Prob>F**	**Remarks**
Model	6807.81	9	756.42	91.38	< 0.0001	Significant
A-catalyst dose	1394.87	1	1394.87	168.51	<0.0001	
B-oxidant dose	1421.97	1	1421.97	171.78	<0.0001	
C-dye conc.	195.80	1	195.80	23.65	0.0007	
AB	330.14	1	330.14	39.88	<0.0001	
AC	5.408E-003	1	5.408E-003	6.533E-004	0.9801	
BC	7.938E-003	1	7.938E-003	9.590E-004	0.9759	
A^2	1905.50	1	1905.50	230.20	<0.0001	
B^2	1892.05	1	1892.05	228.57	<0.0001	
C^2	122.72	1	122.72	14.82	0.0032	
Residual	82.78	10	8.28			
Lack of Fit	48.63	5	9.73	1.42	0.3538	Not significant
Pure Error	34.15	5	6.83			
Cor Total	6890.59	19				
Std. Dev.	2.88		R-squared		0.9880	
Mean	77.26		Adj. R-squared		0.9772	
C.V.	3.72		Pred. R-squared		0.9394	
Press	417.90		Adeq. Precision		27.718	

Final Equation in Terms of Coded Factors (CFA-$ZnFe_2O_4$

$$Degradatio$$
$$=+\ 93.59 + 9.43 \times A + 10.19 \times B - 2.67 \times C$$
$$O.069 \times B \times C - 12.71 \times A^2 - 9.84 \times B^2 - 3.2[illegible]$$

ICE Plots for Predictor Effects

The Individual Conditional Expectation (ICE) plots in the Figure S-1 illustrate how the predictors CFA-ZnF, H_2O_2, and MB individually affect degradation. ICE plots are valuable for visualizing individual predictor relationships with the response variable while considering interactions with other predictors. The ICE plot of CFA-ZnF showed that as the concentration of CFA-ZnF increases, the degradation also increases, presenting a positive correlation. This trend is evident as most individual lines (gray) and the average trend line (red) display an upward trajectory, peaking around a CFA-ZnF value of 10. This means that higher levels of CFA-ZnF are linked to improved degradation efficiency. The grey lines denote the conditional expectation for individual data point in the dataset. Each grey line illustrates how the predicted response (degradation) varies with the value of the corresponding predictor (CFA-ZnF, H_2O_2, or MB) while keeping the rest of the predictors constant. This helps visualize the variability and individual effects of the predictors on the response variable at different points. The red line provides the summary of the overall effect of the predictor. The ICE plot corresponding to H_2O_2, also exhibits a positive correlation with degradation. As the concentration of H_2O_2 increases, degradation increases as well, with a noticeable peak around the H_2O_2 value of 10-15. The consistency of this trend across individual lines and the average trend line highlights the significant role of H_2O_2 in the degradation process. In contrast, the third plot for MB negatively correlates with degradation. As the concentration of MB increases, degradation decreases, which is indicated by the downward trend in both the individual lines and the average trend line. This suggests that higher concentrations of MB inhibit the degradation process. Overall, these ICE plots clearly represent how each predictor individually affects the degradation process. The positive correlations observed for CFA-ZnF and H_2O_2 underscore their critical roles in enhancing degradation, while the negative correlation for MB highlights its inhibitory effect.

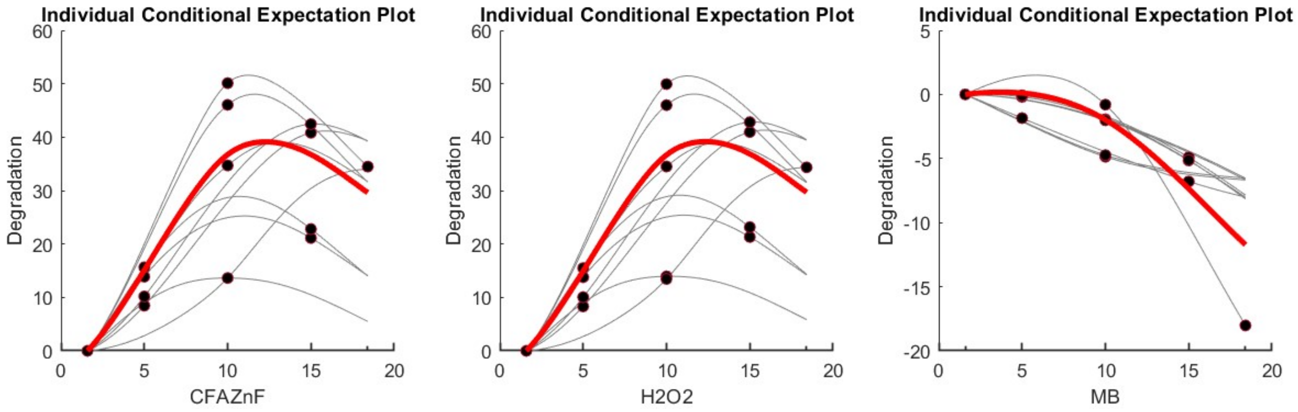

Figure S-1 Individual Conditional Expectation (ICE) Plots for Predictors

Shapley Summary Analysis

The provided Figure S-2 contains two Shapley Summary Plots, which illustrate the importance and effect of each predictor—H_2O_2, CFA-ZnF, and MB—on the degradation process. Shapley values, derived from cooperative game theory, quantify the contribution of each predictor to the model's output. The left plot is a Shapley Summary Plot that highlights individual Shapley values for each predictor across different observations. Each point represents an individual Shapley value, with the color indicating the predictor's value (low to high). For H_2O_2, most Shapley values are positive, suggesting a generally positive impact on degradation. The spread of points along the positive axis indicates varying degrees of contribution, with higher values of H_2O_2 (yellow points) generally associated with higher positive Shapley values. Similarly, CFA-ZnF also shows predominantly positive Shapley values, indicating its positive influence on degradation. The color gradient for CFA-ZnF points shows a trend where higher values of the predictor lead to higher Shapley values. In contrast, MB exhibits mostly negative Shapley values, signifying its inhibitory effect on degradation. The yellow points associated with higher MB values align with more negative Shapley values, reinforcing the negative correlation observed in the previous plots. The right plot is a Shapley Summary Box Plot, which provides a more aggregated view of the distribution of Shapley values for each predictor. For H_2O_2, the box plot shows a wide range of positive Shapley values, with the interquartile range (IQR) lying significantly above zero. This indicates that H_2O_2 consistently contributes positively to degradation across different observations. The CFA-ZnF box plot similarly shows positive Shapley values with a slightly narrower IQR, reinforcing its positive role in degradation. The MB box plot, however, reveals predominantly

negative Shapley values, with the IQR situated below zero. This further substantiates MB's negative influence on the degradation process. Overall, the Shapley Summary Plots provide a detailed and nuanced understanding of each predictor's role. H_2O_2 and CFA-ZnF are consistently shown to positively impact degradation, with their contributions varying across different levels of the predictors. Conversely, MB is confirmed as an inhibitor, with higher values leading to more substantial negative impacts on degradation. These insights are critical for fine-tuning the degradation process and optimizing the conditions to maximize efficiency.

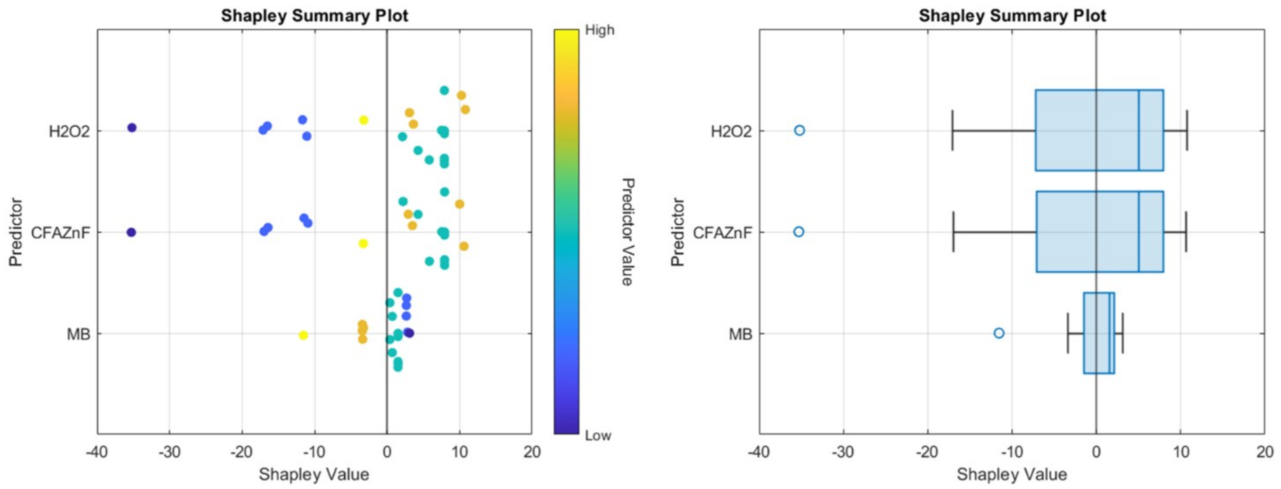

Figure S-2 Shapley Summary Plots for Predictor Contributions

The image displays Local Interpretable Model-agnostic Explanations (LIME) for 20 query points, each illustrating the impact of three predictors (1, 2, and 3) on the response variable. Each bar chart represents the LIME values for a specific query point, indicating the contribution of each predictor to the model's prediction for that point.

1. **Predictors 1 and 2**: Most query points show a strong positive LIME value, suggesting a significant positive influence on the degradation response.
2. **Predictor 3**: Often shows negative LIME values, indicating a negative impact on the response variable.

The consistent pattern across multiple query points highlights the robustness of the predictors' effects, with predictors 1 and 2 generally enhancing degradation, while predictor 3 tends to decrease it. This visualization supports the earlier correlation findings and provides insights into the predictors' roles in the model's decision-making process.

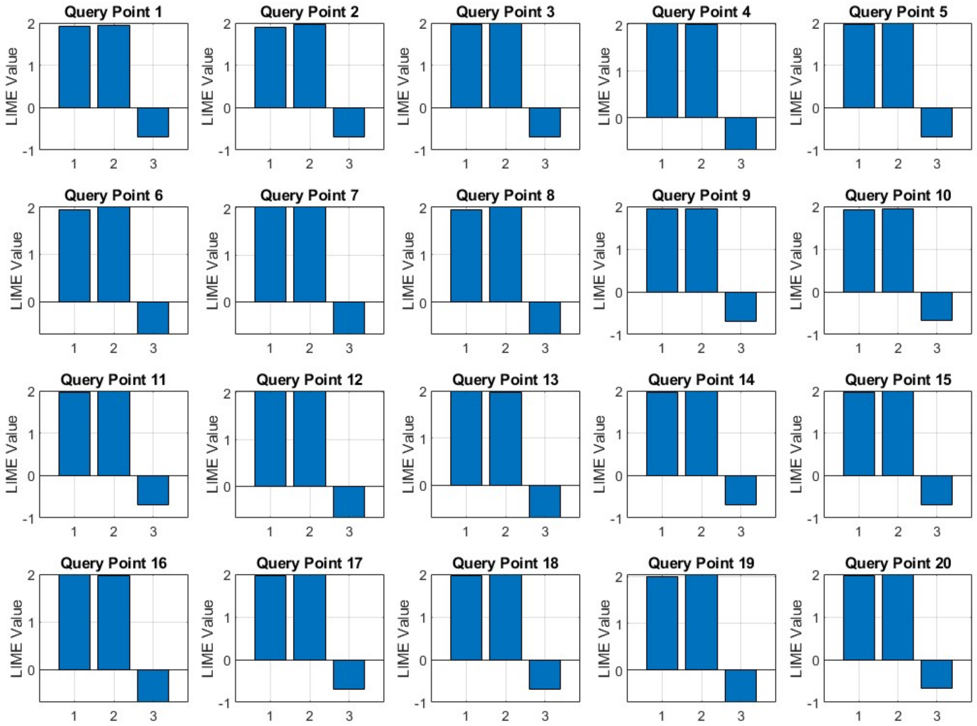

Figure 3 LIME analysis for 20 query points, showing the impact of three predictors on degradation

Predictor Contributions by Interpretability Methods

The provided Figure S-3 illustrates the percentage contribution of three predictors—CFA-ZnF, H_2O_2, and MB—toward the degradation process, evaluated using four different interpretability methods: Shapley Values, Permutation Importance, LIME Values, and F-test Scores. Each method offers a unique perspective on the relative importance of these predictors. The top-left pie chart depicts the contributions based on Shapley Values, a method rooted in cooperative game theory that fairly distributes the total prediction value among the features. According to this chart, CFA-ZnF and H_2O_2 have nearly equal importance, contributing 44.06% and 44.26% respectively, while MB's contribution is significantly lower at 11.68%. In the top-right pie chart, which represents Permutation Importance, the contributions are somewhat different. Here, CFA-ZnF has a slightly higher contribution at 48.71%, followed closely by H_2O_2 at 46.52%. MB again shows the least importance, contributing only 4.77%. This method assesses feature importance by measuring the increase in the model's prediction error when the feature's values are randomly shuffled.

The bottom-left pie chart shows the contributions based on LIME (Local Interpretable Model-agnostic Explanations) Values. This method explains individual predictions by approximating the model locally with an interpretable model. According to this chart, H_2O_2 has the highest contribution at 42.88%, followed by CFA-ZnF at 42.34 %, and MB at 14.78%. Finally, the bottom-right pie chart represents the contributions based on F-test Scores, which evaluate the statistical significance of each feature. Here, H_2O_2 has the highest contribution at 47.99%, followed closely by CFA-ZnF at 47.36%, while MB remains the least important predictor with a contribution of 4.65%. In summary, across all four interpretability methods, H_2O_2 and CFA-ZnF consistently show substantial contributions to the degradation process, with their relative importance varying slightly depending on the method used. MB, on the other hand, consistently appears as the least influential predictor. This consistency across different interpretability methods highlights the robustness of the findings and reinforces the critical roles of CFA-ZnF and H_2O_2 in the degradation process.

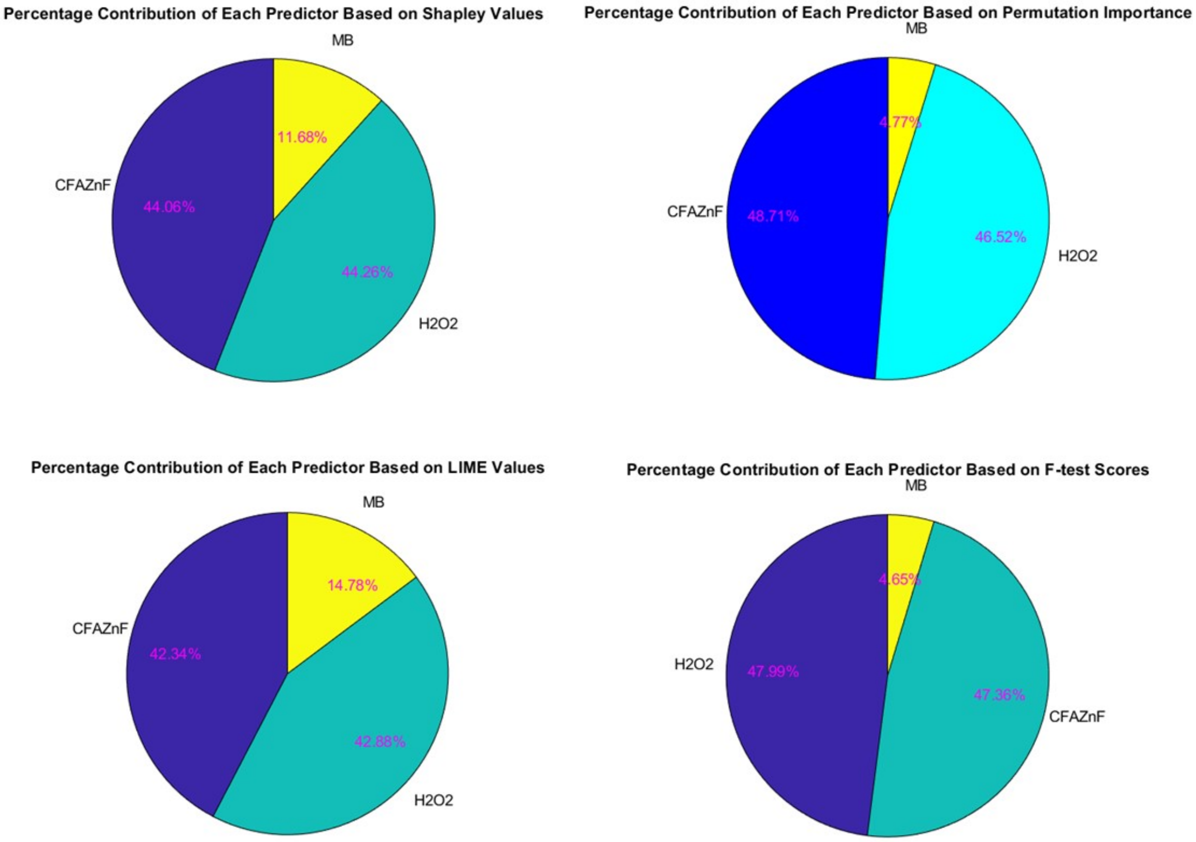

Figure S-4 Percentage Contribution of Each Predictor Based on Different Interpretability Methods

Figure S-5 represents the

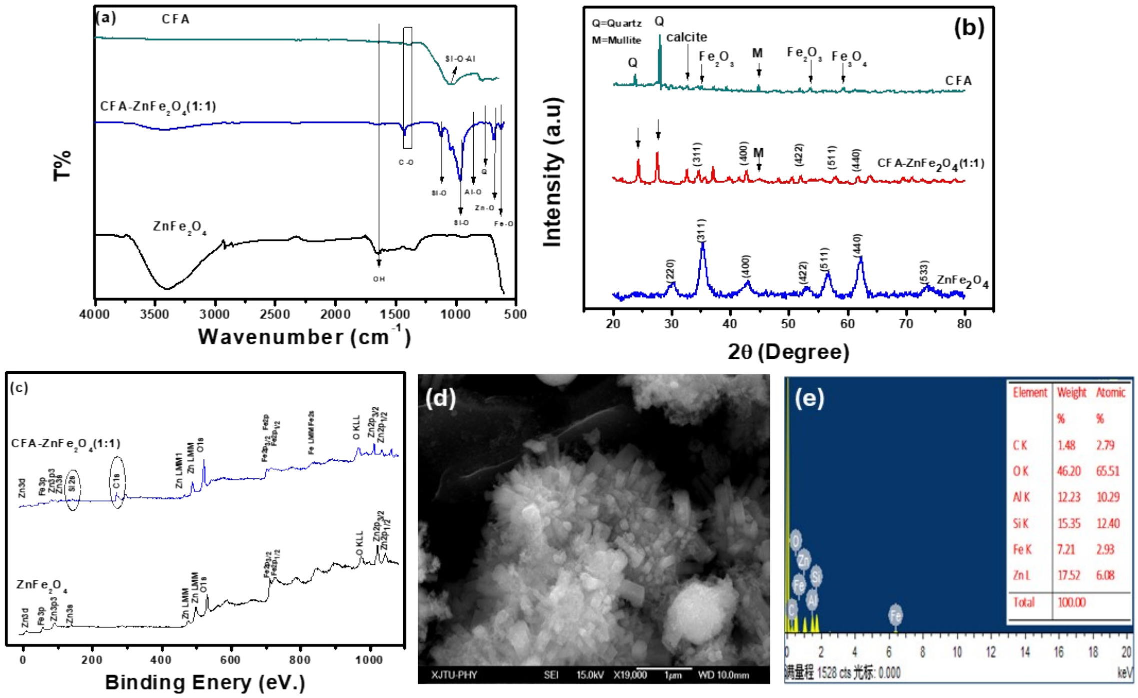

Figure S-5 Characterization analysis (a) FTIR analysis, (b) XRD analysis, (c) XPR survey scans, (d and e) SEM EDX analysis of CFA-ZnF (1:1)
